# Prevalence and phylogenetic analysis of human enteric emerging viruses in porcine stool samples in the Republic of Korea

**DOI:** 10.3389/fvets.2022.913622

**Published:** 2022-09-30

**Authors:** Daseul Yeo, Md. Iqbal Hossain, Soontag Jung, Zhaoqi Wang, Yeeun Seo, Seoyoung Woo, Sunho Park, Dong Joo Seo, Min Suk Rhee, Changsun Choi

**Affiliations:** ^1^Department of Food and Nutrition, Chung-Ang University, Anseong-si, South Korea; ^2^Department of Food and Nutrition, Gwangju University, Gwangju, South Korea; ^3^Division of Food Bioscience and Technology, College of Life Sciences and Biotechnology, Korea University, Seoul, South Korea; ^4^Bio and Environmental Technology Research Institute, Chung-Ang University, Seoul, South Korea

**Keywords:** enteric virus, emerging virus, phylogenetic analysis, enterovirus, adenovirus, foodborne virus, pig

## Abstract

Emerging infectious diseases (EID) in humans and animals are proving to be a serious health concern. This study investigated the prevalence of emerging or re-emerging human enteric viruses in porcine stools and swabs. Eleven enteric EID viruses were selected as target viruses for the current study and ranked based on their impact on public health and food safety: enterovirus (EV), hepatitis E virus, norovirus GI and GII, sapovirus (SaV), adenovirus (AdV), astrovirus, rotavirus, hepatitis A virus, aichivirus, and bocavirus. Using real-time RT-PCR or real-time PCR, EID viruses were detected in 129 (86.0%) of 150 samples. The most prevalent virus was EV, which was detected in 68.0% of samples, followed by AdV with a detection rate of 38.0%. In following sequencing and phylogenetic analyses, 33.0% (58/176) of the detected viruses were associated with human enteric EID viruses, including AdV-41, coxsackievirus-A2, echovirus-24, and SaV. Our results show that porcine stools frequently contain human enteric viruses, and that few porcine enteric viruses are genetically related to human enteric viruses. These findings suggest that enteric re-emerging or EID viruses could be zoonoses, and that continuous monitoring and further studies are needed to ensure an integrated “One Health” approach that aims to balance and optimize the health of humans, animals, and ecosystems.

## Introduction

Emerging infectious diseases (EID) are defined as newly recognized infectious diseases in a community or existing diseases that rapidly increase in incidence or expand in geographic range. Many emerging diseases are zoonotic—the disease-causing organism first incubates in an animal host and then spreads to humans at random (1). Zoonotic diseases spread from animals to humans *via* direct contact, contaminated food and/or water, or the environment, and account for about 61% of the infectious organisms affecting humans (2). Viral pathogens transmitted *via* the fecal-oral route are often reported as EID infectious agents. EID viruses can spread between humans and have pandemic potential, which has recently led to the COVID-19 pandemic (3).

Worldwide, 3,618 cases of EID have been reported from 2016 to 2018 (4), with 331 cases of infectious diseases recorded in the United States during the first 6 months of 2019 (4). The Republic of Korea has noted an increase in EID virus-related deaths between 1996 and 2015, with the rate increasing from 16.5 per 100,000 to 44.6 per 100,000 (5). Recently, EID viruses were identified as the main pathogenic agents in cases of intestinal infections, viral hepatitis, respiratory tract infections, and sepsis (5), and enteric EID viruses were the main cause of disease in such cases. The hepatis A virus (HAV) and the hepatitis E virus (HEV), for example, are representative EID viruses that cause viral hepatitis and intestinal infections, and the HEV and the enterovirus (EV) have become the leading causes of infections in humans overall. As a zoonotic viral disease, numbers of HEV infections escalated from 514 in 2005 to 5,617 in 2015 across Europe (6). Similarly, different serotypes of EV are prevalent at different frequencies in different parts of the world. For example, since the 1980s, seasonal endemic EV-A71 has been prevalent in the U.S., causing small sporadic outbreaks (7). Similarly, EV-A71 continues to cause large epidemics of hand foot and mouth disease (HFMD) and neurological diseases every 1–3 years in the Asian region since the 1990s (7).

There are various causes underlying the emergence of EID viruses, including increasing human and livestock densities, altering patterns of wild-to-domestic animal contact, direct human-to-wild animal contact, and changes in host species diversity (8). As a reservoir of viral EID, pigs represent the major livestock with the most human contact. Various viruses (e.g., picornaviruses, arboviruses, circoviruses, flaviviruses, and herpesviruses) can infect both pigs and humans (9). Because various viruses use pigs as their host, the investigation of the role of pigs as a potential EID virus reservoir is essential to understand the circumstances under which these pathogens emerge and evolve, especially in light of the “One Health” approach that is needed to control zoonoses. However, although research on human enteric viruses has extensively covered waterborne transmission, little information is so far available on enteric EID virus transmission in animals (10). Therefore, we focused on the role of pigs as a potential EID virus reservoir in this study.

The aim of this study was to identify potential enteric EID viruses by examining detection rates and analyzing the genetic relationships between the detected viruses in the Republic of Korea. We selected enteric viruses that are being monitored in eight countries around the world and identified their prevalence in pigs, the livestock closest to humans.

## Materials and methods

### Ethical approval

The study design, animal handling, and experimental protocols were reviewed and approved by the Institutional Animal Care and Use Committee (IACUC) of Chung-Ang University, Republic of Korea (IACUC approval number: CAU2018-00112). All experiments were conducted in accordance with the IACUC guidelines and regulations.

### Selection of enteric EID viruses

**Figure 1:**
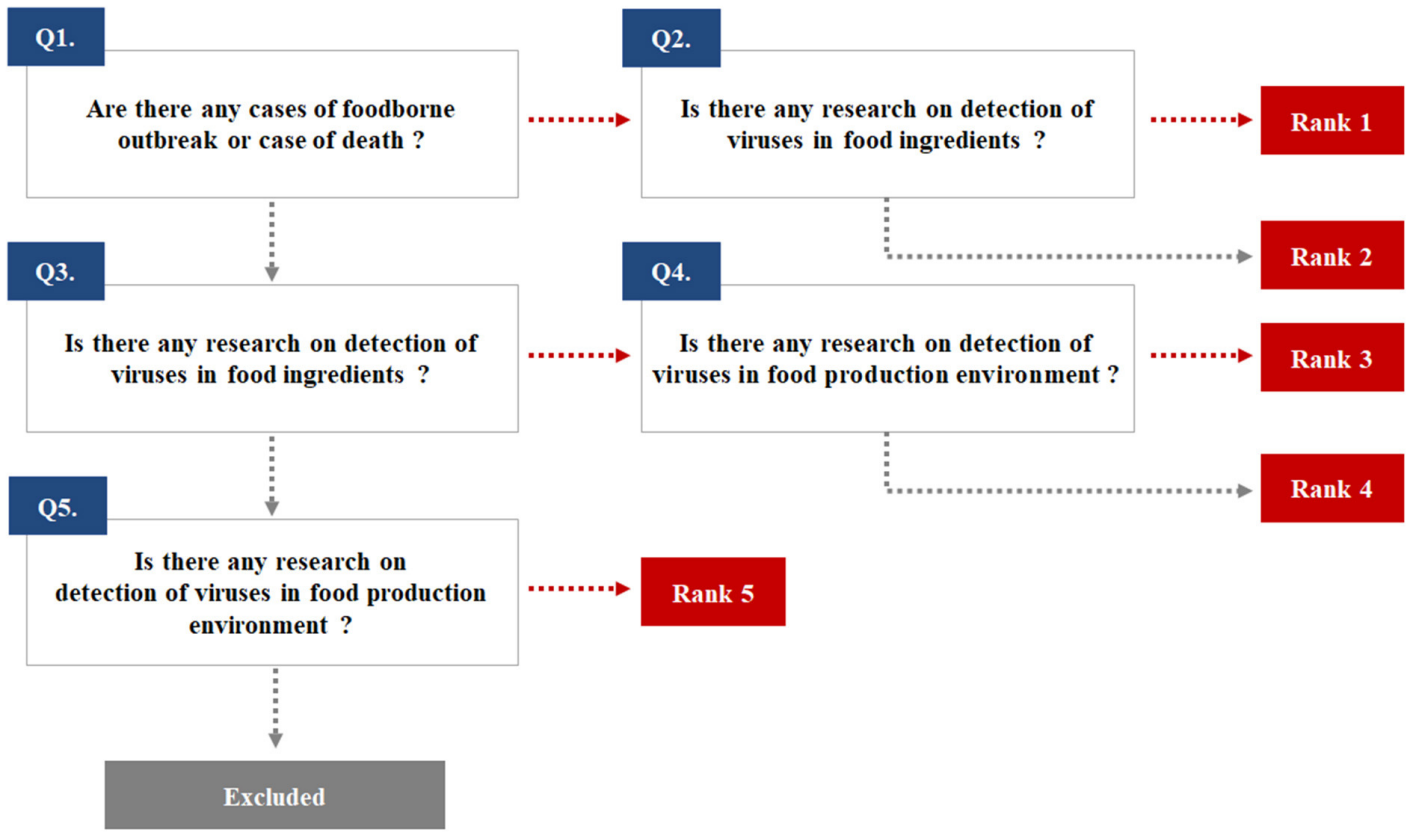
The ranking used to select emerging infectious viruses transmitted through the fecal-oral route.

The potential enteric EID viruses were identified from pathogen lists regulated by the relevant organizations or institutions of each country and from previous publications. A total of 20 human and animal health agencies and food safety administrations were included in our selection process (Supplementary Table 1). Enteric EID viruses were ranked based on their priority for and impact on public health and food safety (Figure 1). The selection of target viruses is depicted in Supplementary Table 2. Following the decision process illustrated in Figure 1, eleven target viruses were selected: norovirus (NoV)-GI, NoV-GII, sapovirus (SaV), HEV, adenovirus (AdV), aichivirus (AiV), astrovirus (AstV), HAV, and rotavirus (RotaV) in rank 1; human bocavirus (BoV) and EV including coxsackievirus (CV), echovirus (EchoV), and poliovirus in rank 3. No enteric EID was classified as rank 2.

### Sample preparation and nucleic acid extraction

One hundred nineteen porcine stool samples and 31 porcine rectal swabs were collected from 14 pig farms located in Gyeonggi-do, Gyeongsang-do, Jeolla-do, and Chungcheong-do provinces in the Republic of Korea. A total of 87 piglets and 63 sows were tested for enteric EID virus prevalence (Table 1). All samples were collected from healthy pigs and transferred to a laboratory for analysis. To minimize sampling stress, veterinarians collected pig stools and rectal swabs from several different pens at each farm, following the IACUC protocol guidelinesWe used the BD CultureSwab MaxV collection and transport systems (Becton, Dickinson and Company, NJ, USA) for rectal swabs and Norgen Biotek's Stool Nucleic Acid Collection and Preservation system (Lubio Science, Zurich, Switzerland) to collect porcine stools. Each sample was composed of 10% (v/v) stool suspension in phosphate-buffered saline (PBS; 0.1 M, pH 7.4). The suspension was prepared as previously described (11). Viral RNA and DNA were extracted using the RNeasy Mini kit (Qiagen, Hilden, Germany) according to the manufacturer's instructions. The extracted RNA and DNA samples were stored at −80°C until real-time reverse transcription PCR (RT-qPCR) or real-time PCR (qPCR) was performed.

**Table 1:** Stages and sample types of 150 pigs at a pig farm in the Republic of Korea.

**Region**	**Stage**		**Total**
** Sample type**	**Sows**	**Piglets**	
Gyeonggi-do	9	11	20
Stools	9	11	20
Gyeongsang-do	47	47	94
Stools	46	19	65
Swabs	1	28	29
Jeolla-do	2	8	10
Stools	2	8	10
Chungcheong-do	5	21	26
Stools	3	21	24
Swabs	2	0	2
Total	**63**	**87**	**150**

### RT-qPCR or qPCR for the detection and titration of enteric EID viruses

The primers and probes used for one-step RT-qPCR and qPCR are presented in Supplementary Table 3. One-step RT-qPCR for detecting each RNA virus was performed with the one-step RT-PCR kit (Qiagen, Hilden, Germany), while qPCR was performed using the Premix Ex Taq (2X)™ kit (Takara, Shiga, Japan) to identify the DNA viruses. RT-qPCR and qPCR were performed on the CFX96™ Real-Time PCR system (Bio-Rad, CA, USA); the titration of each virus sample was determined using either RT-qPCR or qPCR. Synthetic RNA and DNA sequences were used in the assay as standard templates for titration. Synthetic templates, including quantitative synthetic DNA of human AdV-41 (ATCC^®^ VR-930DQ, Virginia, US) and quantitative synthetic RNA of NoV-GII (ATCC^®^ VR-3235SD, Virginia, US), were used as standard templates for AdV and NoV, respectively, and IDT-synthetic RNA oligo (IDT, IA, USA) was used as the standard template for EV, HEV, and RotaV.

For RT-qPCR- or qPCR-positive samples, nested (RT)-PCR was performed to obtain amplicons for sequence analysis. The primers used for nested (RT)-PCR are presented in Supplementary Table 4. Nested (RT)-PCR was performed using a one-step RT-PCR kit (Bioneer, Daejeon, Republic of Korea). Amplicon size was confirmed on 1.0% agarose gel electrophoresis and then used for the sequencing analysis.

### Sequence analysis and phylogenetic tree

Purification of PCR product was performed using a nucleospin gel and a PCR Clean-up Mini Kit (Macherey-Nagel, Düren, Germany). The sequence analysis was performed with capillary electrophoresis on a SeqStudio Genetic Analyzer (Thermo Fisher Scientific, MA, USA). Sequence data were edited using the SeqMan programme (DNASTAR, WI, USA). The sequences were analyzed by comparison with different viral genotype sequences using BLAST and the Norovirus Typing Tool version 2 (https://www.rivm.nl/mpf/typingtool/norovirus/) (12).

To establish the genetic relationships between the detected viruses, phylogenetic analyses were carried out using the nucleotide sequences. During the phylogenetic analysis of the detected EID viruses, with the MEGA X software (http://www.megasoftware.net), the RNA-dependent RNA polymerase (RdRp) gene was targeted for EV, the hexon and fiber genes for AdV, ORF2 and VP1 overlapping regions for HEV, the VP1 region for SaV, and the ORF1-ORF2 junction region for NoV-GII. For the sequencing analysis, a bootstrap consensus tree inferred from 1,000 replicates was taken to represent the evolutionary history of the analyzed taxa. Branches corresponding to partitions reproduced in < 50% of the bootstrap replicates were collapsed (13).

## Results

### Prevalence of EID viruses detected in porcine stools and swabs

One hundred twenty-nine (86.0%) of the 150 samples tested were positive for the presence of at least one EID virus (Table 2). A total of 85 (56.6%) and 44 (29.3%) of the 129 positive samples were identified as containing single and multiple viruses, respectively. In the single virus-containing samples, EV (including EV-G, CV-A2, and EchoV) and AdV were most commonly detected, in 61 and 22 of the 85 samples, respectively. In contrast, 31 of the 44 samples containing multiple viruses tested positive for both EV-G and AdV.

**Table 2:** Profiles of enteric EID viruses detected in porcine stools and swabs.

		**Number of positive samples (%)**		
		**Stools (*n* = 119)**	**Rectal swabs (*n* = 31)**	**Total (*n* = 150)**
**Detected virus type**
**None**		17 (14.3)	4 (12.9)	21 (14.0)
**Single**				
	EV-G	50 (42.0)	5 (16.1)	55 (36.6)
	CV-A2	0 (0.0)	5 (16.1)	5 (3.3)
	EchoV	1 (0.8)	0 (0.0)	1 (0.7)
	AdV	17 (14.3)	5 (16.1)	22 (14.6)
	NoV-GII	1 (0.8)	0 (0.0)	1 (0.7)
	SaV	1 (0.8)	0 (0.0)	1 (0.7)
	**Total**	**70 (58.8)**	**15 (48.3)**	**85 (56.6)**
**Multiple**				
	EV-G + AdV	21 (17.6)	10 (32.3)	31 (20.6)
	EV-G + HEV	4 (3.4)	0 (0.0)	4 (2.6)
	EV-G + SaV	3 (2.5)	0 (0.0)	3 (2.0)
	EV-G + NoV-GII	0 (0.0)	1 (3.2)	1 (0.7)
	CV-A2 + HEV	0 (0.0)	1 (3.2)	1 (0.7)
	EV-G + NoV-GII + AdV	1 (0.8)	0 (0.0)	1 (0.7)
	HEV + AdV	1 (0.8)	0 (0.0)	1 (0.7)
	HEV + AdV + NoV-GII	1 (0.8)	0 (0.0)	1 (0.7)
	HEV + AdV + SaV	1 (0.8)	0 (0.0)	1 (0.7)
	**Total**	**32 (26.9)**	**12 (38.7)**	**44 (29.3)**
**Susceptible host type**
	Human	39 (22.2)	19 (10.8)	58 (33.0)
	Porcine	86 (48.7)	18 (10.2)	104 (59.1)
	Zoonotic	12 (6.8)	2 (1.1)	14 (8.0)
	**Total** ^**¶**^	**137 (77.9)**	**39 (22.2)**	**176**

^¶^Susceptible host classification was based on the total number of detected virus (176) including multiple detection.

Table 2 shows that overall, EV was detected in 102 (68.0%) of the 150 samples, while EV-G was the most prevalent genotype, detected in 95 (93.1%) of the 102 EV-positive samples. AdV, HEV, SaV, and NoV-GII were found in 57 (38.0%), eight (5.3%), five (3.3%), and four (2.7%) out of 150 samples, respectively. Further, HEV was not identified as a single virus in any sample but only detected alongside EV-G, CV-A2, AdV, NoV-GII, and SaV. Moreover, depending on the host tropism characteristics, 33.0% (58/176) of the viruses were determined to be human enteric viruses, including CV-A2, EchoV, AdV, and SaV-GI; in contrast, 59.1% (104/176) and 8.0% (14/176) of the viruses detected in this study were porcine enteric viruses (including EV-G, porcine AdV-5, and porcine AdV-3) and zoonotic enteric viruses (including NoV-GII.11, NoV-GII.18, HEV-GIII, and SaV-GV).

Additionally, the viral loads of EV-G, CV-A2, and EchoV-24 ranged between 1.3 and 6.8, 2.3 and 5.9, and 3.7 log10 genome copies/mL, respectively. The viral loads of AdV, HEV, and NoV-GII ranged between 0.1 and 5.1, 0.5 and 3.5, and 0.6 and 3.0 log10 genome copies/mL (average values, 2.4, 1.9, and 1.3 log10 genome copies/mL). In addition, the viral load of SaV was not determined in this study. Although 82.6% (124/150) porcine samples tested positive for RotaV on RT-qPCR, nested RT-PCR failed to obtain amplicons for sequence analysis. NoV-GI, AstV, HAV, BoV, and AiV were not detected in any of the porcine samples (data not shown).

#### Genotype and phylogenetic analysis of enteric EID viruses detected in porcine samples

##### Enterovirus

**Figure 2:**
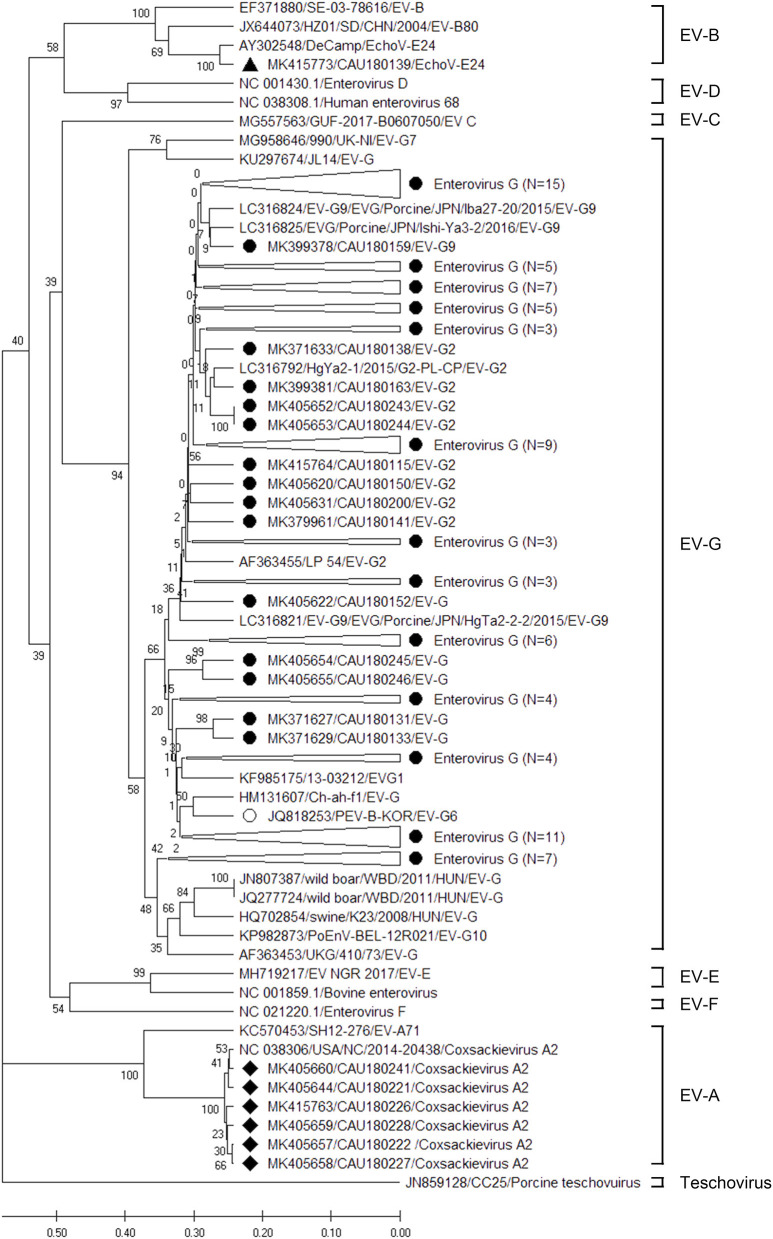
Phylogenetic analysis of the EVs detected in porcine stools and swabs. The EV phylogenetic tree was constructed using the Unweighted Pair Group Method with Arithmetic Mean (UPGMA) and based on the 191-bp sequence of the RdRp region. The black circles (•), black squares (■), and black triangle (▴) indicate the EV-G, CV- A2, and EchoV-E24 sequences detected in this study, respectively. The white circle (°) represents an EV-G (JQ818253/PEV-B-KOR/EV-G6) reference sequence that was reported in the Republic of Korea. The phylogenetic analysis included the EV genotype: EV-A (two strains), EV-B (three strains), EV-C (one strain), EV- D (two strains), EV-G (16 strains), EV-E (two strains), EV-F (one strain), and teschovirus (one strain) as reference sequences. Large triangles (△) on the phylogenetic tree represent the compression of a subtree with a genetic relationship, with the numbers of compressed sequences shown in parentheses. The numbers at the nodes of the tree indicate bootstrap values and the scale bar indicates nucleotide substitutions per site.

Amplicons derived from positive samples were further characterized by sequencing. The phylogenetic analysis of RdRp region fragments for EV is shown in Figure 2. Six EV sequences belonged to the EV-A genogroup and clustered with a reference sequence of NC038306 (CV-A2). The nucleotide sequence identity is shown in Supplementary Table 5, exhibiting a nucleotide sequence identity of 98.8–100.0%. On the other hand, only one EchoV-E24 (MK415773) was identified as belonging to genogroup EV-B, and it clustered with the AY302548 reference sequence, demonstrating 100.0% nucleotide sequence identity. The EV-G genogroup was identified in five genotypes: EV-G1, EV-G2, EV-G6, EV-G9, and EV-G10. Moreover, 53 sequences belonged to a large cluster of EV-G9, which contained reference sequences of LC316821, LC316825, and LC316824, demonstrating a high percentage of nucleotide sequence identity, around 84.1–95.1%, with porcine strain LC316825. Thirteen EV-G2 sequences clustered with porcine reference sequences (LC316792 and AF363455), while their nucleotide sequence identity with LC316792 and AF363455 was 86.6–95.1% and 86.6–91.5%, respectively. Twelve EV sequences clustered with EV-G1 and exhibited 52.4–58.5% nucleotide sequence identity with the KF985175 reference sequence. The ten EV-G6 sequences showed 75.6–89.0% nucleotide sequence identity with the JQ818253 reference sequence, which was not reported on before the Republic of Korea. The seven sequences of EV-G matched with EV-G10. The nucleotide identity of the clusters of EV-G10 with the KP982873 reference sequence was 78.0–82.9%.

##### Adenovirus

**Figure 3:**
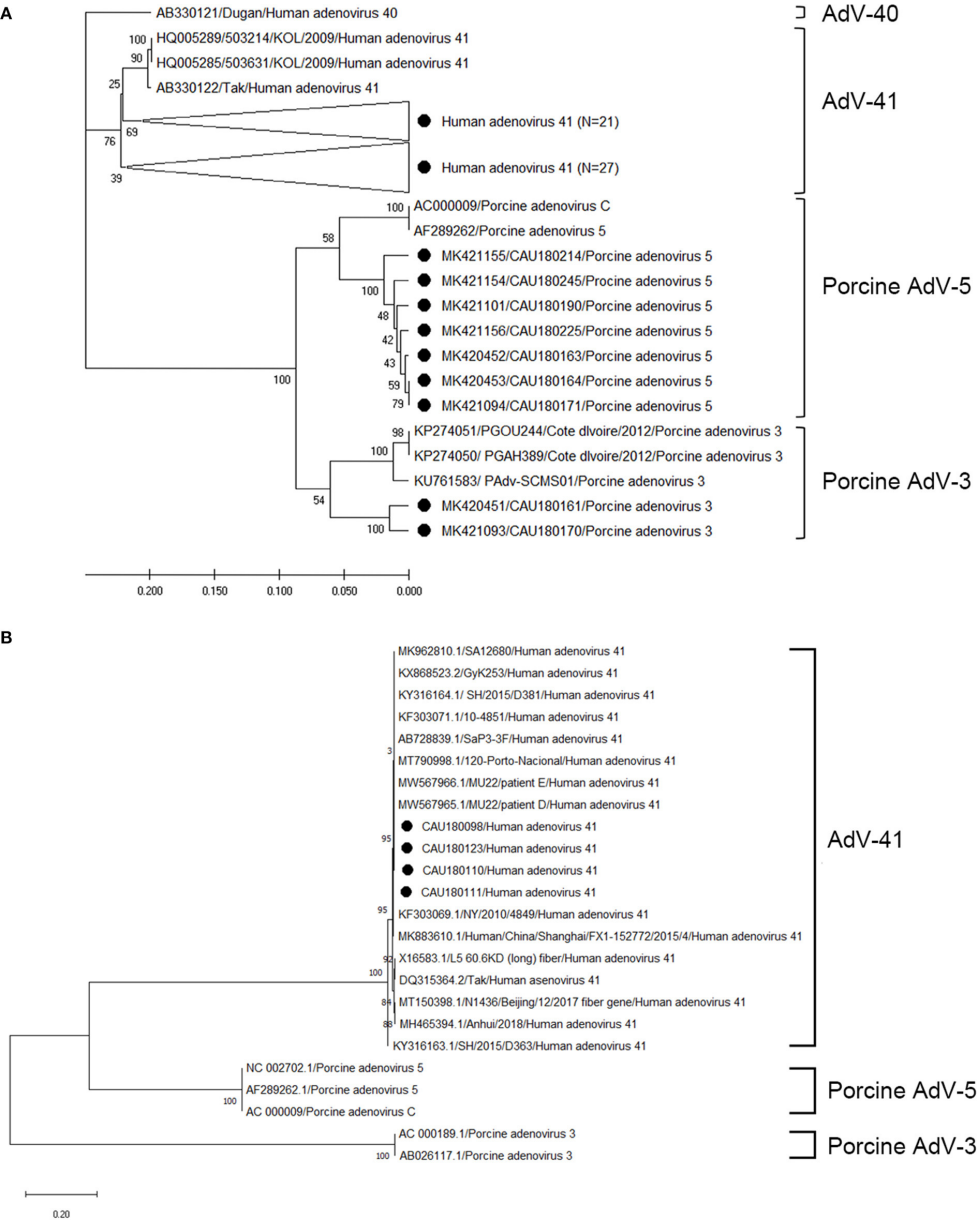
Phylogenetic tree of AdV detected in porcine stools and swabs. Sequences detected in this study are represented as black circles (•). **(A)** Phylogenetic tree analysis for the AdV hexon gene using the UPGMA method and based on the 171-bp sequence. Here, AdV-41 (three strains), AdV-40 (one strain), porcine AdV-5 (two strains), and porcine AdV-3 (three strains) were used as reference sequences. Large triangles (⊲) on the phylogenetic tree represent the compression of a subtree with a genetic relationship, with the numbers of compressed sequences shown in parentheses. **(B)** Phylogenetic tree analysis for AdV (662-bp of the fiber gene) using the maximum likelihood method. Here, AdV-41 (15 strains), porcine AdV-5 (three strains), and porcine AdV-3 (two strains) were used as reference sequences. The numbers at the nodes of the tree indicate bootstrap values and the scale bar indicates nucleotide substitutions per site.

The sequences of AdV positive samples were analyzed for the hexon and fiber genes using a phylogenetic tree as depicted in Figure 3; the nucleotide sequence identities are listed in Supplementary Tables 6, 7. For the hexon gene, our analysis shows that 48, seven, and two AdV sequences clustered with the human AdV-41, porcine AdV-5, and porcine AdV-3 genogroups, respectively (Figure 3A). However, the sequence identity range of the human AdV-41 genogroup with the AB330122 (human AdV-41 Tak strain) reference sequence was 97.3–99.1%; likewise, the porcine AdV-5 genogroup clustered with the AC000009 (porcine AdV C strain) reference sequence and showed 93.8–95.9% nucleotide sequence identity, while the porcine AdV-3 genogroup showed 81.4–83.2% nucleotide sequence identity with the KU761583 (porcine AdV-3 strain) reference sequence. Four sequences of AdV were also analyzed for fiber genes using a phylogenetic tree (Figure 3B), and we found that the resulting sequences clustered with human AdV-41 and exhibited 99.1–99.3% nucleotide sequence identity with the reference sequence DQ315364 (human AdV-41 Tak strain). A comparison of the AdV sequences with those obtained for the hexon gene also confirmed the presence of human AdV-41 strains but not porcine AdV strains.

##### Hepatitis E virus

**Figure 4:**
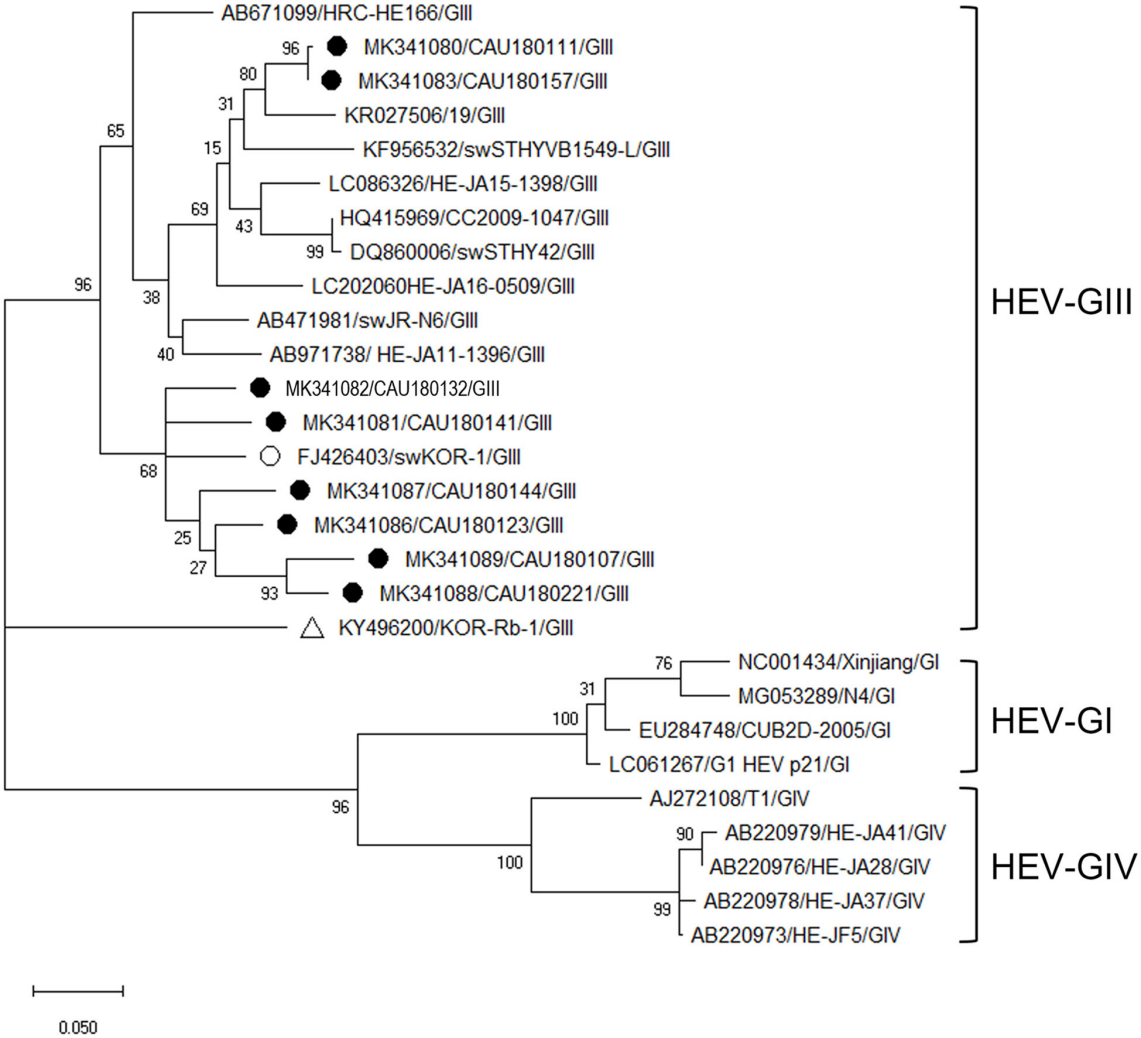
Phylogenetic analysis of HEV detected in porcine stools and swabs. The HEV phylogenetic tree was constructed following the UPGMA method and based on 348-bp sequences of ORF2 and VP1 overlapping region. Sequences detected in this study are represented as black circles (•). The reference sequences of porcine HEV (FJ426403/swKOR-1) and rabbit HEV (KY496200/KOR-Rb-1) strains detected in the Republic of Korea are labeled with white circle (°) and triangle **(Δ)**, respectively. The phylogenetic analysis included HEV-GI (four strains), HEV-GIII (11 strains), and HEV-GIV (five strains) as reference sequences. The numbers at the nodes of the tree indicate bootstrap values and the scale bar indicates nucleotide substitutions per site.

Phylogenetic analysis of HEV was based on ORF2 and VP1 overlapping regions (Figure 4). All investigated HEV sequences in this study were confirmed to belong to the GIII genogroup. During the phylogenetic analysis, eight HEV sequences (MK341089, MK341088, MK341086, MK341081, MK341087, MK341082, MK341080, and MK341083) clustered with the porcine HEV reference sequences (FJ426403 and KR027506). Particularly, the MK341082 sequence showed a high nucleotide sequence identity of 93.9% with the FJ426403 reference sequence (Supplementary Table 8).

##### Sapovirus

**Figure 5:**
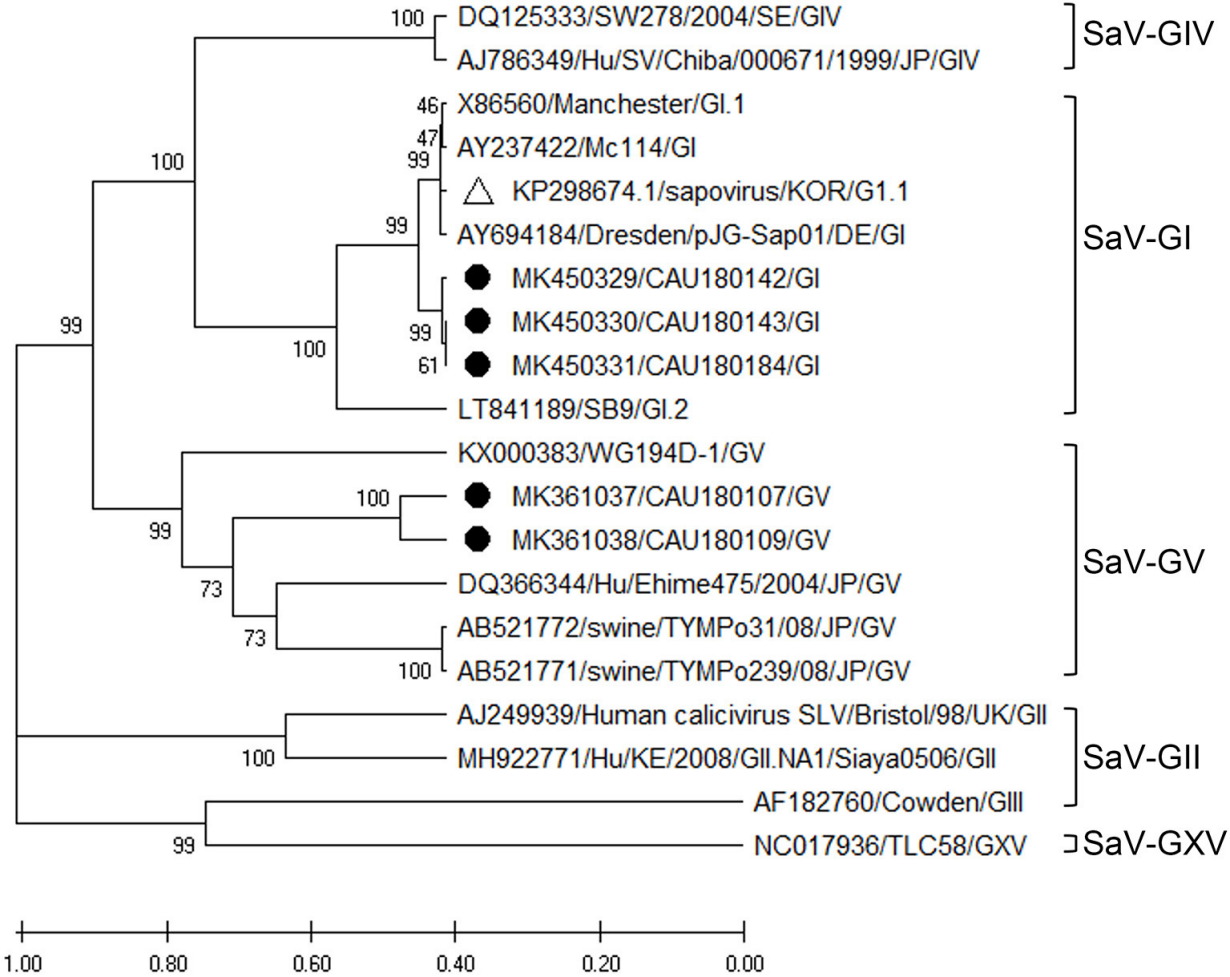
Phylogenetic analysis of SaV detected in porcine stools and swabs. The SaV phylogenetic tree was constructed following the UPGMA method and based on 416-bp sequences of the VP1 region. Sequences detected in this study are represented as black circles (•). The white triangle (Δ) represents the human SaV strain (KP298674/KOR/G1.1) that was confirmed in the Republic of Korea. The phylogenetic analysis included SaV-GI (four strains), SaV-GII (two strains), SaV-GIII (one strain), SaV-GIV (two strains), SaV-GV (four strains), and SaV-GXV (one strain) as reference sequences. The numbers at the nodes of the tree indicate bootstrap values and the scale bar indicates nucleotide substitutions per site.

The phylogenetic analysis of SaV was based on the VP1 region, as depicted in Figure 5, and the nucleotide sequence identities are listed in Supplementary Table 9. According to our analysis, three (MK450329, MK450330, and MK450331) and two (MK361037 and MK36103) sequences of SaV belonged to the GI and GV genogroups, respectively. Moreover, the SaV-GI cluster showed 94.1–94.6% nucleotide sequence identity with the human SaV reference sequences (AY694184 and KP298674), while the SaV-GV cluster exhibited 77.2–77.5% nucleotide sequence identity with the porcine SaV reference sequence (AB521772).

##### Norovirus GII

**Figure 6:**
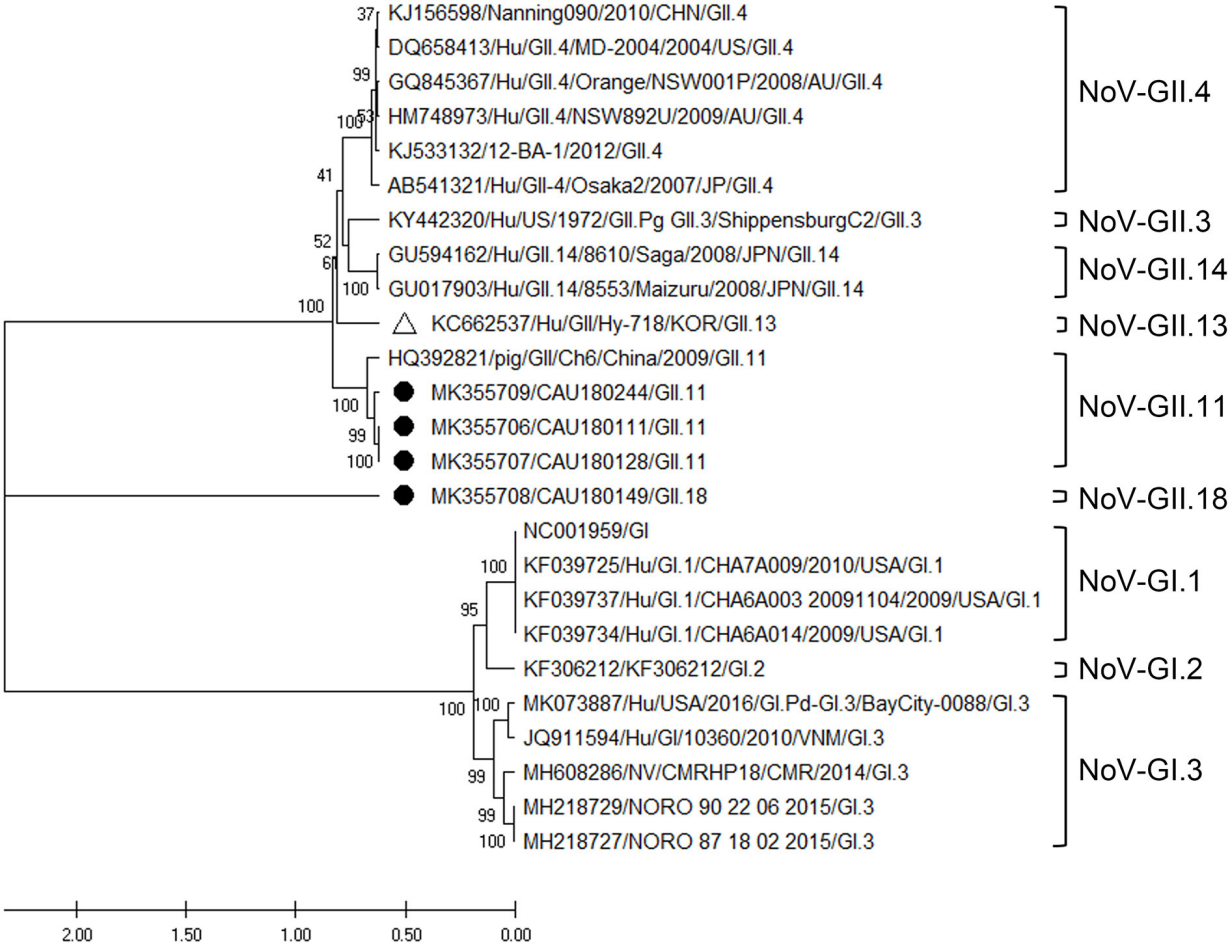
Phylogenetic analysis of NoV detected in porcine stools and swabs. The NoV phylogenetic tree was constructed following the UPGMA method and based on 310-bp sequences of the RdRp region. Sequences detected in this study are labeled with black circles (•). The white triangle (Δ) represents the human NoV strain (KC662537/Hu/GII/Hy-718) that was confirmed in the Republic of Korea. As reference sequences, NoV-GII.4 (six strains), GII.3 (one strain), GII.14 (two strains), GII.13 (one strain), GII.11 (one strain), and GI (10 strains) were used. The numbers at the nodes of the tree indicate bootstrap values and the scale bar indicates nucleotide substitutions per site.

Three NoV-GII.11 and one NoV-GII.18 sequence were confirmed by phylogenetic analysis of the RdRp region, as shown in Figure 6. According to analysis, the three sequences (MK355709, MK355707, and MK355706) belonging to the NoV-GII.11 genotype clustered with a porcine NoV reference sequence (HQ392821). Their nucleotide sequence identity with the HQ392821 reference sequence was 88.2–88.6%, and that with the human NoV reference sequence (KC662537) 72.0–73.3% (Supplementary Table 10).

### Discussion

In this study, eleven enteric EID viruses were chosen based on their priority for and impact on public health and food safety. We observed a high prevalence of enteric EID viruses in porcine samples collected from domestic pig farms in the Republic of Korea. A total of 176 viruses were detected, including several viruses that were detected in multiple cases per same porcine stools and rectal swabs. The positivity rate for EID viruses in individual porcine stools and rectal swabs was 86.0%. As for single viruses, EV and AdV were the most commonly detected viruses in the 150 samples tested. As for multiple detection, 31 of the 44 positive samples were positive for both EV and AdV. We detected human enteric viruses, CV-A2, EchoV, AdV, and SaV-GI, and porcine enteric viruses, EV-G, porcine AdV-5, and porcine AdV-3. Moreover, the zoonotic viruses NoV-GII.11, NoV-GII.18, HEV-GIII, and SaV-GV were detected (representing 9.3% of all detected viruses). The porcine enteric virus detection rate was 69.3% (104/150), with most being EV-G, while human enteric viruses were detected in 38.7% (58/150) of samples. To our knowledge, there is no prior report on the detection of CV-A2, EchoV, AdV-41, and SaV-GI in pigs in the Republic of Korea.

Detection was often prolonged, due to the ability of the viruses to survive on environmental surfaces, in foods, and in water (14). Viruses detected in stools have three possible sources: direct shedding from infected pigs, contaminated pig farm environments, and contaminated workers (15). Regarding the prevalence of enteric EID viruses, porcine stools and rectal swabs showed detection rates of 85.7% (102/119) and 87.0% (27/31), possibly indicating direct virus infection of the animals, although most of the detected viruses were EV and AdV in both sample types. However, among the detected viruses (EV and AdV), AdV-41 and CV-A2 are known to infect only humans. Moreover, enteric viruses were extremely stable on pork chops when stored at low temperatures (16, 17). The infectivity of the viruses was not confirmed since they were only detected *via* PCR, but the presence of human enteric viruses in porcine samples indicates the possibility of the virus spreading to animals, thereby providing a possible cause of zoonotic infection. In addition, a virus can be transmitted from an infected pig to humans through direct contact with the environment or contaminated instruments as well as through the consumption of contaminated undercooked meat (18).

Our study based on phylogenetic analyses evaluated the genotypes and sub-genotypes of enteric EID viruses in porcine stools and swabs. The RdRp genomic region that was analyzed for EV is a highly conserved genomic region, according to previous reports (19). During our phylogenetic analysis of the detected EV, three EV genogroups were identified, which belonged to EV-A, EV-B, and EV-G. Within these genogroups, EV-A included six sequences of CV-A2 detected on porcine rectal swabs only, with a high nucleotide sequence identity (98.8–100.0%) with the NC038306 (CV-A2) reference sequence discovered in the US (20). CV is one of the major public health problems among children in the Republic of Korea, with ~214,642 (0.53%) of 40,461,309 outpatients surveilled in a study from 2010 to 2013 diagnosed with CV-induced HFMD (21). In addition, the EV-B genogroups show frequent recombination within species (22), and one sequence of EchoV-E24 (MK415773) that was detected in porcine stool clustered with the AY302548 (EchoV-E24) reference sequence isolated from humans in the US in 2004 (22). This indicates that the capsid region of the corresponding EchoV-E24 (MK415773) sequence also needs to be analyzed for more accurate genotyping. In the Republic of Korea, no cases of infection or hospitalization due to EchoV-E24 have been reported, but one out of 579 HFMD pediatric patients is diagnosed with an EchoV-E24 infection in China in 2010 (23). Moreover, the EV-G genogroup consists of three large clusters. Among them, the largest clade contains 89 sequence groups with EV-G1, EV-G2, EV-G6, and EV-G9, while the EV-G10 group includes seven sequence groups. Reports from Japan and Germany have stated that 51 sequences belong to a large cluster of EV-G9 (24, 25). To date, the EV-G genogroup consists of 17 types of genotypes from EV-G1 to EV-G17 (25), of which EV-G1, EV-G2, EV-G6, EV-G9, and EV-G10 were identified in this study. In the Republic of Korea, EV-G6 (JQ818253) was first reported in 2009, also isolated from porcine stool (26). According to our findings, 11 sequences of EV-G6 clustered with the JQ818253 strain, exhibiting a 75.6–89.0% nucleotide sequence identity. The other 84 sequences' average nucleotide sequence identity with the JQ818253 strain was 80.9%. This is presumed to be from a similar era than the ancient JQ818253 strain. For a higher phylogenetic resolution, the capsid region of VP1 also needs to be analyzed in future studies.

The sequences positive for AdV were analyzed for hexon and fiber genes. The hexon gene is highly conserved (27), making it the best single region of the AdV genome for genus-specific detection; other major capsid protein regions were identified to target the fiber gene that was used for the subgroup determinant validation. Our phylogenetic analysis of the detected AdV revealed that the sequences belonged to the human AdV-41, porcine AdV-5, and porcine AdV-3 genogroups. An additional analysis of some fiber genes confirmed a higher sequence identity with the AdV reference sequences, of 99.1–99.3%, as that reported in Asian countries (28). Porcine AdV infections were usually asymptomatic, but some cases of mild diarrhea or mild respiratory signs in porcine have been reported (29); however, no signs of pig infections were recorded in this study. In particular, in view of the potential application of porcine AdV as virus vectors for vaccines and the use of animal AdV as vectors for gene therapy, the results of this study indicate that attention should be paid to health and infectious disease management and vaccine development.

The HEV prevalence rate varies by country, region, and even farms within a country (30). Our phylogenetic analysis of all detected HEV sequences belonging to the same genotype, HEV-GIII, showed a high nucleotide identity (93.9%) with FJ426403 (swine HEV isolate swKOR-1). Meanwhile, the six HEV-positive sequences found in this study clustered with sub-genotype GIIIa containing reference strain FJ426403 (31), while two other positive sequences (MK341080, MK341083) were grouped with KR027506, which has been found to infect humans in France, belonging to the sub-genotype GIIIc (32). However, HEV-positive sequences belonging to sub-genotype GIIIa were detected at higher rates than GIIIc, in line with reports that the HEV-GIIIa sub-genotype is predominantly prevalent in the Republic of Korea and Japan while the GIIIc sub-genotype is predominantly prevalent in Europe (32). Likewise, our study also confirmed the genetic association of the detected HEV to infect both humans and porcine HEV. In the Republic of Korea, the sub-genotype GIIIa detected in cats and oysters was found to be genetically close to porcine and human HEV (31). According to Wilhelm et al. (33), HEV sequences identified in Canadian retail pork livers closely match human strains. It is widely known that HEV is an emerging zoonotic agent and that pigs represent an important reservoir (31). Moreover, ingestion of HEV-contaminated raw or undercooked pig products is the main source of HEV transmission through food (34). Therefore, to prevent indigenous human HEV infections in the Republic of Korea, one should be careful about coming in contact with infected animals and consuming contaminated meat.

SaV has been detected in humans, pigs, mink, dogs, sea lions, bats, chimpanzees, and rats (25, 35). The SaV genome contains two overlapping open reading frames (ORFs): ORF1 and ORF2. ORF1 encodes the non-structural proteins and the capsid protein, VP1, while ORF2 encodes the minor structural protein, VP2. Furthermore, SaV is genetically highly diverse and classified into nineteen genogroups based on the VP1 sequences (35, 36). Among the nineteen SaV genogroups, four genogroups (GI, GII, GIV, and GV) and eight genogroups (GIII, GV, GVI, GVII, GVIII, GIX, GX, and GXI) have been found to be linked to human and pig infections, respectively (36). However, as far as we are aware, this is the first time that the SaV-GV genogroup (in two detected sequences, MK361037 and MK36103) has been detected in porcine stools in the Republic of Korea. In addition, based on our phylogenetic analysis, the SaV-GV genogroup was more closely related to porcine SaV than human SaV. Accordingly, the SaV-GI genogroup clusters with the KP298674 reference strain isolated from human stools (37). Overall, detected isolates and reference strains differ between porcine and human stool, and it is presumed that there may be genetic differences, even within the same GI genogroup.

The NoV genus covers viruses that infect a variety of hosts such as humans, pigs, cattle, and mice, and this broad range of hosts provides the opportunity for its zoonotic spread (38). Among the various hosts, asymptomatic infected pigs are known as natural reservoirs of NoV. Moreover, porcine NoV is genetically most similar to human NoV. In addition, NoV-GII.11 and GII.18 genotypes have been reported as sources of infections in pigs (39). Accordingly, porcine NoV has been mainly detected in pigs (40). Among the diverse range of human NoV genotypes in GII, NoV-GII.18 shares the highest amino acid identity with the human GII.3 (39). Our study results also show the genetic similarity of NoV-GII.18 (MK355708) to human NoV-GII.4. Furthermore, in the Republic of Korea, the NoV-GII.11 was detected in fecal samples from asymptomatic food handlers (41). According to a phylogenetic analysis, the MK355709, MK355707, and MK355706 sequences cluster with the HQ392821 reference sequence isolated from pigs with diarrhea symptoms in China (40). These results indicate that porcine NoV has a high potential for zoonotic transmission of enteric EID.

Several enteric viruses are very similar to human viruses found in pigs, and some are thought to have zoonotic potential. In this study, all of the target viruses were detected, which shows how common they are in Korean pig populations (42–44). Sequence analyses were conducted to determine how likely these viruses are to spread to humans. However, only few positive RT-qPCR samples could be used for conventional RT-PCR and sequencing, potentially due to the fact that the assays have different sensitivities and we could therefore not successfully amplify the samples using conventional RT-PCR. However, it cannot be ruled out that the primer systems we used were not able to detect certain viruses. Therefore, RT-PCR systems that can reliably identify different porcine viruses should be designed in the future. Our sequence analysis shows that the few viruses that are typical to pigs are only weakly related to human strains. Another result of our phylogenetic analysis is that pig and human strains were grouped together on different branches of the phylogenetic trees.

The emergence of enteric EID viruses exemplifies the complex interaction of humans, animals, and the environment (45). Many viruses have been shown to survive for a long period in their natural reservoirs (46), and viruses are constantly spreading from natural hosts to humans and other animals (47), due to human activities such as modern agriculture and urbanization (48). Keeping in mind the “One Health” philosophy, the best strategy to prevent viral zoonosis is to maintain the natural viral reservoirs separate from human society. Zoonotic agents have been responsible for the majority of current human health threats (49). One of the most important facets of public health is veterinary public health (VPH), where veterinarians are responsible for safeguarding animal and human health and wellbeing. Viral pathogenesis studies for domestic and wild animals, as well as for human diseases, are coordinated by veterinary virologists working in VPH. This coordination is essential to our understanding of how viruses spread and affect individual health and populations over time; it is also important for preparing for the emergence of new human diseases. However, veterinarians must be aware of the disease's prevalence, risk factors, control strategies, and associated costs and benefits in order to adequately advise producers on disease management. Good hygienic practices during slaughtering are required to reduce the danger of these viruses being introduced into the food chain. Furthermore, highly exposed individuals, such as slaughterers and veterinarians, should be made more aware of the need to prevent direct transmissions.

In further studies, the prevalence and zoonotic potential of all pig viruses suspected to infect humans should be assessed using larger samples and including more geographical regions to precisely evaluate the risk of zoonotic virus transmission. Furthermore, whole-genome sequence analyses are needed for a more comprehensive approach, rather than genotyping of specific genes.

### Conclusion

This study documents the molecular detection and diversity of human enteric EID viruses in porcine stools and swabs collected from pig farms in the Republic of Korea. Our results indicate that human enteric viruses detected in pigs and some porcine enteric viruses are genetically related to human enteric viruses. In addition, the previously known zoonotic viruses, such as HEV-GII, NoV-GII, SaV, EV, and AdV, were detected in porcine samples, indicating the zoonotic potential of porcine enteric viruses as potential EIDs.

### Data availability statement

The datasets presented in this study can be found in online repositories. The names of the repository/repositories and accession number(s) can be found in the article/Supplementary material.

### Ethics statement

The study design, animal handling, and experimental protocols were reviewed and approved by the Institutional Animal Care and Use Committee (IACUC) of Chung-Ang University, Republic of Korea (IACUC approval number: CAU2018-00112). All experiments were conducted in accordance with the IACUC guidelines and regulations. Written informed consent was obtained from the owners for the participation of their animals in this study.

### Author contributions

DS, MR, and CC: conceptualization and supervision. DY, MH, SJ, and ZW: validation. DY and SJ: formal analysis and software. DY, SJ, ZW, YS, SW, and SP: investigation and methodology. DY, MH, and SJ: visualization. DY, DS, MR, and CC: project administration. DY, SJ, and SP: data curation. CC: resources. DY: writing—original draft preparation. DY, MH, SJ, and CC: writing—review and editing. MR and CC: funding acquisition. All authors have read and agreed to the published version of the manuscript.

## Funding

This research was supported by a grant (17162MFDS034) from the Ministry of Food and Drug Safety in 2017-2019. YS was supported by the Chung-Ang University Graduate Research Scholarship in 2021.

## Conflict of interest

The authors declare that the research was conducted in the absence of any commercial or financial relationships that could be construed as a potential conflict of interest.

## Publisher's note

All claims expressed in this article are solely those of the authors and do not necessarily represent those of their affiliated organizations, or those of the publisher, the editors and the reviewers. Any product that may be evaluated in this article, or claim that may be made by its manufacturer, is not guaranteed or endorsed by the publisher.

## Supplementary material

Supplementary Table 1 List of institutions reviewed for selection of enteric EID.

Supplementary Table 2 Rank of emerging infectious viruses.

Supplementary Table 3 Primers used for the screening of target viruses.

Supplementary Table 4 Primers used for the sequencing analysis.

Supplementary Table 5 Comparison of nucleotide sequence identity of EV.

Supplementary Table 6 Comparison of nucleotide sequence identity of the AdV hexon gene region.

Supplementary Table 7 Comparison of nucleotide sequence identity of the AdV fiber gene region.

Supplementary Table 8 Comparison of nucleotide sequence identity of HEV.

Supplementary Table 9 Comparison of nucleotide sequence identity of SapoV.

Supplementary Table 10 Comparison of nucleotide sequence identity of NoV.

Supplementary Table 11 GenBank accession numbers for all viral sequences confirmed in this study.
