# Supplementary material for: Prevalence and phylogenetic analysis of human enteric emerging viruses in porcine stool samples in the Republic of Korea
Source: Front Vet Sci. 2022 Sep 30;9:913622. doi: 10.3389/fvets.2022.913622 (PMC9563253; doi:10.3389/fvets.2022.913622)
Supplement: Supplementary Table 1 — List of institutions reviewed for selection of enteric EID. [file Data_Sheet_1.docx]

**Supplementary Table 1**. List of institutions reviewed for selection of enteric EID.

| Country | Human Health | Animal Health | Food Safety |
| --- | --- | --- | --- |
| US | Centers for Disease Control and Prevention (CDC) | Department of Agriculture National Animal Disease Centre (USDA NADC) | Food and Drug Administration (FDA) |
| Republic of Korea | Korea Centers for Disease Control and Prevention (KCDC) | Ministry of Agriculture, Food and Rural Affairs (MAFRA) | Ministry of Food and Drug Safety (MFDS) |
| Germany | Robert Koch Institute (RKI) | Friedrich Loeffler Institute (FLI) | Bundesinstitut fur Risikobewertung (BfR) |
| UK | Health Protection Agency (HPA) | Pirbright Institute | Food Standards Agency (FSA) |
| EU | European Centre for Disease Prevention and Control (ECDC) | Animal Health | European Food Safety Authority (EFSA) |
| Australia and  New Zealand | Australian Infection Control Association (AICA) | Australian Animal Health Laboratory (AAHL) | Food Standards Australia New Zealand (FSANZ) |
| Japan | National Institute of Infectious Diseases (NIID) | National Institute of Animal Health (NIAH) | Ministry of Health, Labour and Welfare (MHLW) |

**Supplementary Table 2.1**Rank of emerging infectious viruses

| **Rank** | **Virus** | | | | | | | | | | | | | | | | |
| --- | --- | --- | --- | --- | --- | --- | --- | --- | --- | --- | --- | --- | --- | --- | --- | --- | --- |
| **Rank 1** | |  | | | | |  | | | |  | | |  | | | |
| Adenovirus | | | Hepatitis E virus | | | | | Hepatitis A virus | | | | | Rotavirus | | | | Astrovirus |
| Aichivirus | | | Sapovirus | | | | |  | | | | |  | | | |  |
| **Rank 2** | | | | | |  | | | | | | | | |  | | |
| Not applicable | | |  | | | | |  | | | | |  | | | |  |
| **Rank 3** | | | | |  | | | | | | |  | | | | | |
| Bocavirus | | | Enterovirus | | | | | Coxsackievirus | | | | | Echovirus | | | | Poliovirus |
| **Rank 4** | | | | |  | | | | | | |  | | | | | |
| Influenza A virus (H1N1) | | | Influenza A virus (H5N1) | | | | | Influenza A virus (H3N2) | | | | | Influenza A virus (H5N2) | | | | Influenza A virus (H5N3) |
| Influenza A virus (H5N8) | | | Influenza A virus (H5N9) | | | | | Influenza A virus (H7N9) | | | | | Encephalitis virus | | | | Nipah virus |
| Avian leukosis virus type-1 | | | Avian paramyxovirus serotype-1 | | | | | Influenza A virus (H5N6) | | | | |  | | | |  |
| **Rank 5** | | | | |  | | | | | | |  | | | | | |
| Human Papillomavirus | | | Coxsackievirus-16 | | | | | Enterovirus-71 | | | | | Mardivirus | | | |  |
| **Excluded** | |  | | | | |  | | | |  | | |  | | | |
| Flavivirus | | | | West Nile virus | | | | | | Lyssavirus | | | | | | Lassa fever virus | |
| Crimean Congo hemorrhagic fever virus | | | | Hantavirus | | | | | | La Crosse virus | | | | | | Puumala virus | |
| Middle East respiratory syndrome | | | | Severe Acute Respiratory Syndrome Coronavirus | | | | | | Ebolavirus | | | | | | Marburg virus | |
| Tick borne meningoencephalitis virus | | | | Japanese encephalitis virus | | | | | | Saint Louis encephalitis virus | | | | | | Hepatitis C virus | |
| Zika virus | | | | Hepatitis B virus | | | | | | Herpesvirus | | | | | | Herpesvirus-1 | |
| Herpesvirus-4 | | | | Herpesvirus-5 | | | | | | Herpesvirus-6 | | | | | | Herpesvirus-7 | |
| Hendra virus | | | | Measles virus | | | | | | Morbillivirus | | | | | | Severe fever with thrombocytopenia syndrome virus | |
| Respiratory syncytial virus | | | | Parvovirus-B19 | | | | | | Enterovirus-D68 | | | | | | Rhinovirus | |
| Orthopoxvirus | | | | Parapoxvirus | | | | | | Vaccinia virus | | | | | | Variola major virus | |
| Human Immunodeficiency virus | | | | Human T-cell lymphotropic virus | | | | | | Bat lyssavirus | | | | | | European bat lyssavirus | |
| Chikungunyavirus | | | | Eastern Equine encephalitis virus | | | | | | Equine encephalitis virus | | | | | | Venezuelan Equine encephalitis virus | |
| Sindbis virus | | | | Togavirus | | | | | | Hepatitis D virus | | | | | | Mumps virus | |
| Vesicular stomatitis virus | | | | Melaka virus | | | | | | Molluscipoxvirus | | | | | | Paramyxovirus | |
| Influenza B virus | | | | Western Equine encephalitis virus | | | | | | Rubella virus | | | | | | Rabies virus | |
| Variola virus | | | | Cowpox virus | | | | | | Parainfluenza viruses | | | | | | Herpesvirus-8 | |
| Herpesvirus-2 | | | | Usutu virus | | | | | | Dengue fever virus | | | | | | Rift valley fever virus | |
| Variegated squirrel bornavirus-1 | | | | Coronavirus | | | | | | Yellow fever virus | | | | | | Herpesvirus-3 | |
| Early summer meningoencephalitis virus | | | | | | | | |  | | | | | |  | | |

| **Virus** | **Primer** | **Sequence (5´-3´) ^§^** | **Reference** |
| --- | --- | --- | --- |
| Enterovirus | EV1 | GAT TGT CAC CAT AAG CAG C | (1) |
|  | EV2 | CCC CTG AAT GCG GCT AAT C |  |
|  | Ev-Probe | CGG AAC CGA CTA CTT TGG GTG TCC GT |  |
| Norovirus-GI | COG1F | CGY TGG ATG CGN TTY CAT GA | (2) |
|  | COG1R | CTT AGA CGC ATC ATC ATT YAC |  |
|  | RING1 | AGA TYG CGA TCY CCT GTC CA |  |
| Norovirus-GII | BPO-13 | ANC CNA TGT TYA GIT GGA TGA G | (3) |
|  | BPO-13N | AGT CAA TGT TTA GGT GGA TGA G |  |
|  | BPO-14 | TCG ACG CCA TCT TCA TTC ACA |  |
|  | BPO-18 | CAC RTG GGA GGG CGA TCG CAA TC |  |
| Hetatitis E virus | JHEV-F | GGT GGT TTC TGG GGT GAC | (4) |
|  | JVHEV-R | CGA AGG GGT TGG TTG GAT G |  |
|  | JHEV-P | ATT CTC AGC CCT TCG CAA TCC CCT |  |
| Adenovirus | JTVFF | AAC TTT CTC TCT TAA TAG ACG CC | (5) |
|  | JTVFR | AGG GGG CTA GAA AAC AAA A |  |
|  | JTVFP | CTG ACA CGG GCA CTC TTC GC |  |
| Aichivirus | Forward | CCC AGT GTG CGT AAC CTT CT | (6) |
|  | Reverse | GTA CCT GCC TGG CAT YCC TA |  |
|  | Probe | ACG CCC TGT GCG GGA TGA AA |  |
| Astrovirus | AstVF | CCD GCC AGR CTC ACA GAA GAG | (7) |
|  | AstVR | GAC TTG CTA GCC ATC ACA CTY C |  |
|  | Probe | ACT CCA TCG CAT TTG GAG GGG AGG ACC |  |
| Rotavirus | NVP3-FDeg | ACC ATC TWC ACR TRA CCC TC | (7) |
|  | NVP3-R1 | GGT CAC ATA ACG CCC CTA TA |  |
|  | NVP3-Probe | ATG AGC ACA ATA GTT AAA AGC TAA CAC TGT CAA |  |
| Bocavirus | Forward | AGA GGC TCG GGC TCA TAT CA | (8) |
|  | Reverse | CAC TTG GTC TGA GGT CTT CGA A |  |
|  | Probe | AGG AAC ACC CAA TCA RCC ACC TAT CGT CT |  |
| Hepatitis A virus | Forward | GCG GCG GAT ATT GGT GAG | (9) |
|  | Reverse | CAA TGC ATC CAC TGG ATG AGA |  |
|  | Probe | TTA AGA CAA AAA CCA TTC AAC GCC GGA G |  |

**Supplementary Table 3**. Primers used for the screening of target viruses.

^§ Using the primer labeled with FAM-TAMRA probe.^

| **Virus** | | **Target gene** | **Primer** | **Sequence (5´-3´)** | **Reference** |
| --- | --- | --- | --- | --- | --- |
| Enterovirus | RdRp | RPOL-1S | YGA RGC NWS NAG YYT NAA YGA | (10) |  |
|  |  | RPOL-1A | AWR TTR TTR ATC ATW GAR TTR AAN AT |  |  |
|  |  | RPOL-2S | WGC MTT TGA YTA YWC NGG NTA YGA YGC |  |  |
|  |  | RPOL-2A | RGT GCC WGA NCA NCC NGA KGG CAT |  |  |
| Norovirus-GII | | ORF1-ORF2 junction | GII-FIM | GGG AGG GCG ATC GCA ATC T | (11) |
|  |  |  | GII-RIM | CCR CCN GCA TRN CCR TTR TAC AT |  |
|  |  |  | GII-F3M | TTG TGA ATG AAG ATG GCG TCG ART |  |
| Sapovirus | | VP1 | SV-F11 | GCY TGG TTY ATA GGT GGT AC | (12) |
|  |  |  | SV-R1 | CWG GTG AMA CMC CAT TKT CCA T |  |
|  |  |  | SV-F21 | ANT AGT GTT TGA RAT GGA GGG |  |
|  |  |  | SV-R2 | GWG GGR TCA ACM CCW GGT GG |  |
| Hepatitis E virus | | ORF2-VP1 overlapping | 3156 | AAY TAT GCM CAG TAC CGG GTT G | (13) |
|  |  |  | 3157 | CCC TTA TCC TGC TGA GCA TTC TC |  |
|  |  |  | 3158 | GTY ATG YTY TGC ATA CAT GGC T |  |
|  |  |  | 3159 | AGC CGA CGA AAT YAA TTC TGT C |  |
| Adenovirus | | Hexon | hex1deg | GCC SCA RTG GKC WTA CAT GCA CAT C | (14) |
|  |  |  | hex2deg | CAG CAC SCC NCG RAT GTC AAA |  |
|  |  |  | nehex3deg | GCC CHY GCM ACN GAI ACS TAC TTC |  |
|  |  |  | nehex4deg | CCY ACR GCC AGN GTR WAN CGM RCY TTG TA |  |
|  |  | Fiber | FiFL | TAT GGA CTT AGG AGA CGG T | (10) |
|  |  |  | FiFR | CGT TCA TTA TTT CGA ACG C |  |
| Rotavirus | | VP7 | VP7-F | ATG TAT GGT ATT GAA TAT ACC | (8) |
|  |  |  | VP7-R | CTA TAC TCT ATA ATA AAA AGC T |  |
|  |  |  | aBT1 | CAA GTA CTC AAA TCA ATG ATG G |  |
|  |  |  | aCT2 | CAA TGA TAT TAA CAC ATT TTC TGT G |  |
|  |  |  | aET3 | ACG AAC TCA ACA CGA GAR G |  |
|  |  |  | aDT4 | CGT TTC TGG TGA GGA GTT G |  |
|  |  |  | aAT8 | GTC ACA CCA TTT GTA AAT TCG |  |
|  |  |  | aFT9 | CTT GAT GTG ACT AYA AAT AC |  |

**Supplementary Table 4**. Primers used for sequencing analysis

**Reference**

1. Monpoeho S, Dehee A, Mignotte B, Schwartzbrod L, Marechal V, Nicolas JC, et al. Quantification of Enterovirus RNA in Sludge Samples Using Single Tube Real-Time RT-PCR. *Biotechniques.* (2000) 29(1):88-93.

2. Loisy F, Atmar R, Guillon P, Le Cann P, Pommepuy M, Le Guyader F. Real-Time RT-PCR for Norovirus Screening in Shellfish. *J Virol Methods*. (2005) 123(1):1-7.

3. Yoo JE, Lee C, Park S, Ko G. Evaluation of Various Real-Time Reverse Transcription Quantitative PCR Assays for Norovirus Detection. *J Microbiol Biotechnol*. (2017) 27(4):816-24.

4. Jothikumar N, Cromeans TL, Robertson BH, Meng X, Hill VR. A Broadly Reactive One-Step Real-Time RT-PCR Assay for Rapid and Sensitive Detection of Hepatitis E Virus. *J Virol Methods.* (2006) 131(1):65-71.

5. Ko G, Jothikumar N, Hill VR, Sobsey MD. Rapid Detection of Infectious Adenoviruses by Mrna Real-Time RT-PCR. *J Virol Methods.* (2005) 127(2):148-53.

6. Nielsen ACY, Gyhrs ML, Nielsen LP, Pedersen C, Böttiger B. Gastroenteritis and the Novel Picornaviruses Aichi Virus, Cosavirus, Saffold Virus, and Salivirus in Young Children. *J Clin Virol*. (2013) 57(3):239-42.

7. Shin H, Park H, Seo DJ, Jung S, Yeo D, Wang Z, et al. Foodborne Viruses Detected Sporadically in the Fresh Produce and Its Production Environment in South Korea. *Foodborne Pathog Dis.* (2019) 16(6):411-20.

8. Lu X, Chittaganpitch M, Olsen SJ, Mackay IM, Sloots TP, Fry AM, et al. Real-Time PCR Assays for Detection of Bocavirus in Human Specimens. *J Clin Microbiol*. (2006) 44(9):3231-5.

9. Di Pasquale S, Paniconi M, Auricchio B, Orefice L, Schultz AC, De Medici D. Comparison of Different Concentration Methods for the Detection of Hepatitis a Virus and Calicivirus from Bottled Natural Mineral Waters. *J Virol Methods*. (2010) 165(1):57-63.

10. Madisch I, Harste G, Pommer H, Heim A. Phylogenetic Analysis of the Main Neutralization and Hemagglutination Determinants of All Human Adenovirus Prototypes as a Basis for Molecular Classification and Taxonomy. *J Virol*. (2005) 79(24):15265-76.

11. Lee SG, Lee SH, Park SW, Suh CI, Jheong WH, Oh S, et al. Standardized Positive Controls for Detection of Norovirus by Reverse Transcription PCR. *Virol J*. (2011) 8(1):1-8.

12. Okada M, Shinozaki K, Ogawa T, Kaiho I. Molecular Epidemiology and Phylogenetic Analysis of Sapporo-Like Viruses. *Arch Virol*. (2002) 147(7):1445-51.

13. Meng XJ, Purcell RH, Halbur PG, Lehman JR, Webb DM, Tsareva TS, et al. A Novel Virus in Swine Is Closely Related to the Human Hepatitis E Virus. *Proc Natl Acad Sci USA*. (1997) 94(18):9860-5.

14. Allard A, Albinsson B, Wadell Gr. Rapid Typing of Human Adenoviruses by a General Pcr Combined with Restriction Endonuclease Analysis. *J Clin Microbiol*. (2001) 39(2):498-505.
